# RCSB PDB web portal RCSB.org for biotechnology research: exploration and visualization of plastic-degrading enzymes at the atomic level in 3D

**DOI:** 10.3389/fbioe.2026.1947029

**Published:** 2026-10-05

**Authors:** Alison Biester, Christine Zardecki, Yana Rose, Joan Segura, Jose M. Duarte, Stephen K. Burley

**Affiliations:** 1 Research Collaboratory for Structural Bioinformatics Protein Data Bank, Rutgers, The State University of New Jersey, New Brunswick, NJ, United States; 2 Institute for Quantitative Biomedicine, Rutgers, The State University of New Jersey, New Brunswick, NJ, United States; 3 Research Collaboratory for Structural Bioinformatics Protein Data Bank, San Diego Supercomputer Center, University of California, La Jolla, CA, United States; 4 Rutgers Cancer Institute, The State University of New Jersey, New Brunswick, NJ, United States; 5 Department of Chemistry and Chemical Biology, Rutgers, The State University of New Jersey, New Brunswick, NJ, United States; 6 Rutgers Artificial Intelligence and Data Science (RAD) Collaboratory, Rutgers, The State University of New Jersey, New Brunswick, NJ, United States

**Keywords:** pairwise structure alignment, plastic-degrading enzymes, protein data bank, protein structure, sequence annotations in 3D, structural analysis, structure search

## Abstract

**Introduction:**

The Research Collaboratory for Structural Bioinformatics Protein Data Bank (RCSB PDB) develops resources for biomolecular structure exploration and analysis that are used by an interdisciplinary community of bio scientists, researchers, and students numbering in the many millions worldwide. The RCSB.org website and suite of APIs provide access to more than 250,000 experimentally determined structures archived in the PDB, making it an invaluable tool for biotechnology, such as research on plastic-degrading enzymes. Engineering plastic-degrading enzymes represents a promising strategy for mitigating environmental accumulation of polyethylene terephthalate (PET).

**Methods:**

In recent years, RCSB.org has provided access to more than 1 million Computed Structure Models (CSMs) alongside experimentally determined structures and introduced new tools for 3D Motif Search, Visualizing Sequence Annotations in 3D, and Pairwise Structure Alignment.

**Results and discussion:**

To identify site-directed mutagenesis targets for improving PET-degrading enzyme efficiency, biotechnology researchers utilize 3D structure data freely available from the PDB. This article describes how RCSB PDB tools for Advanced Search, Visualizing Sequence Annotations in 3D, and Pairwise Structure Alignment can be used to study plastic-degrading enzyme structure and function.

## 1 Introduction

For nearly 55 years, the Protein Data Bank (PDB) has served as the single global repository for experimentally determined 3D atomic level structures of biomolecules and their complexes with one another and small-molecule ligands (wwPDB consortium, 2019). Over this time, PDB data were contributed by ∼60,000 structural biologists (Depositors) using various experimental structure-determination methods (e.g., macromolecular crystallography (MX), 3D electron microscopy (3DEM), nuclear magnetic resonance, and integrative hybrid methods) to study the shapes of important biological macromolecules at the atomic level. The PDB archive currently holds more than 250,000 3D biostructures that have been expertly curated and rigorously validated based on community guidelines. Managed by the Worldwide Protein Data Bank (wwPDB) partnerships (Berman et al., 2003), wwPDB promotes the FAIR (Findable, Accessible, Interoperable, and Reusable) (Wilkinson et al., 2016) and FACT (Fairness, Accuracy, Confidentiality, and Transparency) (van der Aalst et al., 2017) principles of scientific data management. All data are provided at no charge with no limitations on usage.

Knowing the 3D structure of a biological macromolecule is essential for understanding its role in human and animal health and disease, its function in plants and food and energy production, and its importance to other topics related to global prosperity and the bioeconomy. PDB data are used in a wide range of studies, including fields related to fundamental biology, biomedicine, energy sciences, and biotechnology (Feng et al., 2020; Basner, 2017; Markosian et al., 2018).

The Research Collaboratory for Structural Bioinformatics (RCSB) PDB (Burley et al., 2025; Berman et al., 2000; Burley et al., 2022) is the United States data wwPDB center for PDB data. It provides open access to data through the research-focused RCSB.org web portal and APIs (Rose et al., 2021; Piehl et al., 2025), and offers a variety of tools that support searching, browsing, visualizing, validating, and analyzing macromolecular structures. RCSB.org is updated weekly with 400–500 newly released structures combined with annotations of PDB structures from ∼50 trusted external resources.

Meticulous curation of PDB data has enabled developments in Artificial Intelligence (AI) and Machine Learning (ML) by providing carefully and expertly curated training datasets for developing protein structure prediction and design methods. Since 2022, RCSB.org has delivered open access to ∼1 million predicted Computed Structure Models (CSMs) (Burley et al., 2023) from AlphaFold Protein Structure Database (AlphaFoldDB) (Varadi et al., 2024) and RoseTTAFold (from the ModelArchive) (Baek et al., 2021; Tauriello et al., 2025) alongside the PDB archive to provide expanded availability of 3D biostructure data to the global research and education communities. These CSMs represent powerful tools for furthering structural biology; they are also being used to support the design of proteins with biochemical functions not previously seen in nature. CSMs can also be used in enzyme engineering to determine target positions for site-directed mutagenesis. When CSMs are returned in search results, they are distinguished from experimental structures with a cyan computer icon. Prior to using a CSM as a basis for hypothesis generation, it is recommended that users review the predicted local-distance difference test (pLDDT) score and proceed with caution at low confidence levels: very high confidence (pLDDT >90), confident (90 > pLDDT >70), low confidence (70 > pLDDT >50), very low confidence (pLDDT <50) (Tunyasuvunakool et al., 2021). Additional information on CSMs is available in the help documentation and on PDB-101 (Zardecki et al., 2022; Burley et al., 2023). RCSB PDB tools for search, analysis, and visualization can help identify and create opportunities for making advances in biotechnology.

A major area of research in biotechnology is the development of novel and efficient approaches for processing and recycling plastic materials. The first man-made plastic, cellulose nitrate or Parkesine, was created and patented by Alexander Parkes in 1856, but it took many years before plastics rose to prominence as the everyday material it is in the modern era. During World War II, there was a need to conserve natural resources, leading to prioritization of synthetic resource development. It is estimated that plastic production increased by 300% during World War II when plastics were used for making parachutes, ropes, tents, and automobile tires (Nicholson and Leighton, 1942). With plastics established as an inexpensive alternative to wood, paper, metal, and glass, production continued to soar after World War II, with production reaching 2 million tons/year by 1950 (Ritchie and Roser, 2018). During the 1960s, people began to appreciate the environmental liabilities of plastic use when plastic debris was first observed in the world’s oceans (Kenyon and Kridler, 1969). In response to growing environmental concerns, municipalities began adopting recycling mandates in the 1980s. Over recent decades, plastic use has continued to grow, with production rates surpassing 400 million tons/year by 2020. However, recycling plastics has proven to be an expensive task, and, as a result, only 9% of plastics have been recycled, creating a major plastic waste management problem (Geyer et al., 2017).

Large plastic debris along with microscopic plastic fragments, termed microplastics (Thompson et al., 2004), contaminate marine (Thompson et al., 2004; Wagner et al., 2014) and terrestrial (Rillig, 2012) habitats. The majority of plastics are composed of a hydrocarbon backbone, rendering them recalcitrant to biodegradation. Polyethylene terephthalate (PET) has proven to be a notable exception; its polyester backbone is susceptible to depolymerization by microbial hydrolases (Müller et al., 2005; Yoshida et al., 2016; Wei and Zimmermann, 2017; Buchholz et al., 2022). Several types of hydrolases, such as cutinases (Müller et al., 2005; Silva et al., 2005; Sulaiman et al., 2012; Carniel et al., 2017), lipases (Carniel et al., 2017; Kim and Song, 2006; Brackmann et al., 2023), and esterases (Yoshida et al., 2016; Ribitsch et al., 2011; Hu et al., 2010; Ribitsch et al., 2012) act on PET. All known PET-degrading enzymes are serine hydrolases with an α/β-hydrolase fold, including a conserved Ser–His–Asp catalytic triad (Buchholz et al., 2022; Han et al., 2017; Joo et al., 2018). These enzymes can break PET down into component monomers, known as mono (2-hydroxyethyl) terephthalic acid (MHET), which can either be repolymerized or further degraded by other microbial enzymes.

PET-degrading enzymes clearly represent a promising tool for industrial plastic degradation, but barriers remain for using these biocatalysts at scale. Depolymerization of an aromatic polyester such as PET is a challenging biocatalytic reaction because aromatic groups can impart crystallinity, thereby increasing the melting temperature of the enzyme substrate (Marten et al., 2005). Above the glass transition temperature of PET (∼65 °C), it is much more challenging for enzymes to act on the PET polyester bond because the polymer remains in a glassy state (Chen et al., 1998). However, contact with water has been shown to decrease the glass transition temperature of PET to ∼40 °C (Tarazona et al., 2022). Below the glass transition temperature, the polymer becomes more flexible, increasing access to the PET polyester bond.

Use of PET-degrading enzymes gained traction upon discovery of a bacterial species, *Piscinibacter sakaiensis*, that can degrade and bio-assimilate PET (Yoshida et al., 2016). (N.B.: This organism was originally named *Ideonella sakaiensis* and was later reclassified as *Piscinibacter sakaiensis*.) Following discovery of *P. sakaiensis*, increased interest in plastic-degrading enzymes spurred creation of online biological data resources compiling and annotating data concerning known plastic degrading enzymes. PlasticDB (Gambarini et al., 2022) and PAZy (Buchholz et al., 2022) are two such resources, each of which provides information about microbial enzymes degrading various types of plastic, including but not limited to PET-degrading enzymes.

The eubacterium *P. sakaiensis* stands out because it can degrade and bio-assimilate PET and harbors the PET hydrolase (PETase) known to be active at ambient temperatures. It has been hypothesized that the *P. sakaiensis* PETase (PsPETase) can catalyze PET degradation while PET is in the glassy state (Yoshida et al., 2016). This PETase, however, is heat-labile and begins losing activity above 40 °C. Several PsPETase variants have been generated to improve the enzyme activity and thermostability including ThermoPETase (Son et al., 2019), DuraPETase (Cui et al., 2021), FAST-PETase (Lu et al., 2022), and Hot-PETase (Bell et al., 2022). Engineered thermostable variants have achieved melting temperatures as high as 83.5 °C (Lu et al., 2022). Among wild-type PET-degrading enzymes, the leaf-branch compost cutinase (LCC) is the most thermostable known PET-degrading enzyme (Sulaiman et al., 2012). Wild-type LCC maintains its enzyme activity up to ∼70 °C (Yoshida et al., 2016), making it another intriguing target for biotechnology applications. To optimize PET-degrading enzymes for biotechnology applications, knowledge of features driving catalytic activity and thermal stability is required. Herein, we present how RCSB.org tools enable structure search, visualization, and analysis of PET-degrading enzymes. These tools support advances in plastic-degrading biotechnologies by aiding in target identification for rational protein design.

## 2 Methods

The wwPDB policy states that data files contained in the PDB archive are available under the CC0 1.0 Universal (CC0 1.0) Public Domain Dedication. RCSB PDB software products are distributed under the terms of an open source license (MIT or Apache 2.0).

### 2.1 RCSB.org advanced search

Identification of a protein of interest cannot rely solely on basic keyword-matching searches, as proteins with overlapping functions may be described by different names and functional annotations, and evolving classification schemes can further limit search accuracy. To fully capture relevant protein structures found in the PDB, a combination of multiple search approaches is necessary, based on sequence, structure, and functional features.

The RCSB.org Advanced Search interface is accessible through the RCSB.org web portal at rcsb.org/search/advanced (Rose et al., 2026). The tool is implemented as a modular query builder that translates user-defined search criteria into structured requests submitted to the RCSB PDB Search API (search.rcsb.org). Individual query nodes correspond to searches over different data domains, including textual annotations, sequence similarity, three-dimensional (3D) structure similarity, and sequence and 3D motif searches. Query nodes can be combined using Boolean operators (AND, OR), enabling construction of complex multi-criteria searches.

By default, Advanced Search queries experimentally determined structures archived in the PDB. The interface also supports inclusion of CSMs as searchable entries, allowing users to retrieve and analyze experimentally determined structures and computational models within a single query, expanding access to structurally characterized proteins beyond the experimentally determined PDB archive.

### 2.2 Sequence annotations in 3D

Sequence Annotations in 3D (Segura et al., 2022) is an open-source package (github.com/rcsb/rcsb-saguaro-3d) that facilitates the exploration and mapping of biochemical annotations from linear sequences onto 3D macromolecular structures. The package is integrated into the RCSB.org web portal, allowing users to easily visualize positional features in both sequence and structural contexts.

### 2.3 Pairwise structure alignments

The Pairwise Structure Alignment tool enables users to perform 3D superposition of multiple protein structures onto a reference structure, facilitating systematic comparison of global fold conservation, local conformational differences, and active site geometry (Bittrich et al., 2024). This tool supports alignment of multiple protein chains to a reference chain using established structural alignment algorithms (jFATCAT, jCE, jCE-CP, Smith–Waterman 3D (Lafita et al., 2019), and TM-align (Zhang and Skolnick, 2005)). User-available alignment algorithms include both rigid body alignment methods (jFATCAT (rigid), jCE, TM-align, and Smith–Waterman 3D) and flexible alignment methods (jFATCAT (flexible) and jCE-CP). The rigid body alignment methods are well suited for proteins that are closely evolutionarily related and that have similar shapes, whereas the flexible alignment methods are well suited for proteins that are distantly evolutionarily related or that adopt different conformational states (Ma and Wang, 2014). This tool is particularly useful for exploring structural mechanisms underlying enzyme function and for assessing the agreement between experimentally determined structures and CSMs.

The Pairwise Structure Alignment application (rcsb.org/alignment) is implemented as a web-based interface integrated with the RCSB.org web portal. User requests submitted through the web interface are processed through the RCSB PDB Alignment API (alignment.rcsb.org), a programmatic RESTful service that performs the calculations of structural alignments. The API accepts PDB identifiers or custom user coordinates as inputs and returns alignment results, including residue-level correspondences, alignment scores, root-mean-square deviation (RMSD) values, transformation matrices, and superposed coordinates in machine-readable formats. These results are used by the web application to provide interactive visualization and analysis of aligned structures. The Pairwise Structure Alignment web application source code is publicly available through the RCSB PDB GitHub repository (github.com/rcsb/rcsb-pecos-app).

## 3 Results

### 3.1 Finding PET-degrading enzymes with RCSB PDB advanced search

The RCSB.org Advanced Search interface (rcsb.org/search/advanced) provides wholesale access to search tools optimized to query metadata annotations, polymeric sequences, 3D geometry, or functional motifs and combine these queries within a single workflow (Figure 1A). Such complex queries are matched against the entire PDB archive, and users can optionally include CSMs (Figure 1B, lower right) to increase structural coverage across different proteomes, ensuring that homologous, predicted 3D biostructures are also captured (Burley et al., 2023).

**FIGURE 1:**
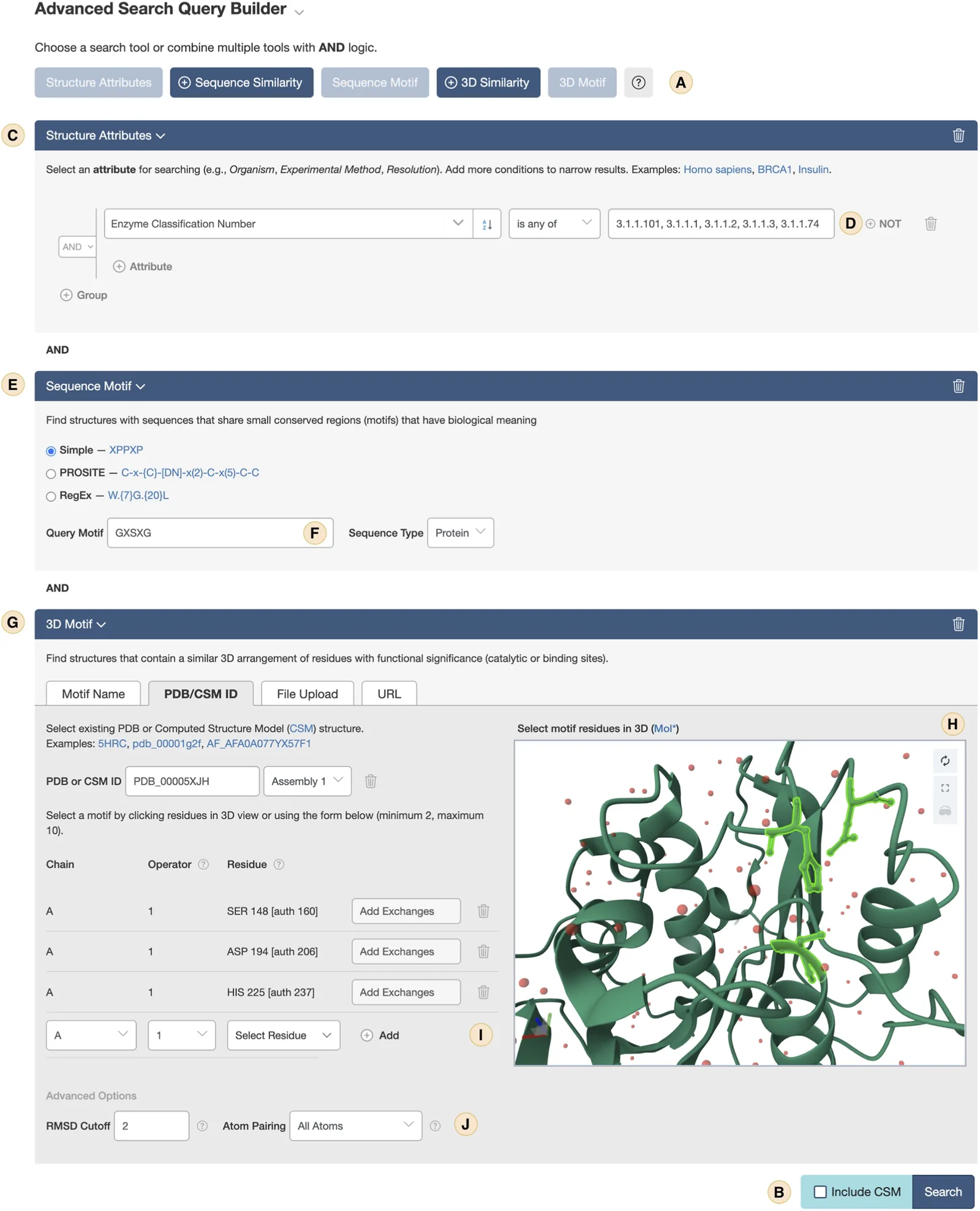
RCSB.org Advanced Search interface identification of PET-degrading enzymes in the PDB archive. **(A)** Advanced Search interface enables selection and combining of multiple search tools, each purpose-built to query a specific type of data (e.g., structured metadata, sequences, or 3D coordinates). **(B)** The “Include CSM” control allows users to include available CSMs alongside experimentally determined structures in the search results. **(C)** Structure Attributes Search panel providing access to curated PDBx/mmCIF metadata and integrated biological annotations. **(D)** Example query using Enzyme Classification (EC) numbers to retrieve PET hydrolases (EC 3.1.1.101) and related carboxylic ester hydrolases (EC 3.1.1.1, 3.1.1.2, 3.1.1.3, 3.1.1.74). **(E)** Sequence Motif Search interface. **(F)** Simple mode query using the conserved GXSXG motif characteristic of serine hydrolases to identify polymer sequences containing the catalytic serine signature. **(G)** 3D Motif Search interface for defining spatial residue arrangements. **(H)** Example selection of catalytic triad residues in the Mol* 3D viewer using PETase from *Piscinibacter sakaiensis* (PDB ID: pdb_00005xjh (Joo et al., 2018)). **(I)** Residue selection panel used to define the Ser–His–Asp catalytic triad as a structural query. **(J)** Advanced Options including RMSD Cutoff, set to the default of 2 Å, and Atom Pairing, set to the default of “All Atoms”.

The RCSB.org Structure Attributes Search (Figure 1C) provides an entry point for query construction, allowing users to explore structured metadata derived from rigorous definitions in the PDBx/mmCIF dictionary (Westbrook et al., 2022), alongside value-added annotations integrated from ∼50 trusted external biological resources. Among these attributes, Enzyme Classification (EC) numbers (Figure 1D) provide a standardized way to filter entries based on annotated catalytic activity, making them a practical starting point for identifying PET-degrading enzymes. Specifically, PET hydrolases can be retrieved by constructing a query that includes their assigned EC number (3.1.1.101), with ECs for related carboxylic ester hydrolases, including lipases and cutinases (e.g., 3.1.1.1, 3.1.1.2, 3.1.1.3, 3.1.1.74), to capture functionally similar enzymes.

To further refine search results, a sequence-based filter can be added. A conserved GXSXG motif is a hallmark of serine hydrolases, identifying the location of the catalytic serine residue within the α/β hydrolase fold (Lord et al., 2013). RCSB.org Sequence Motif Search (Figure 1E) enables detection of such conserved sequence features, and in Simple mode, the motif GXSXG can be directly entered as a query (Figure 1F) to identify matching polymer sequences.

Additional refinement using 3D Motif Search (Figure 1G), targeting the structure of the Ser–His–Asp catalytic triad in three-dimensions, can further focus results on structurally validated PET hydrolases. Within the Ser–His–Asp triad, each residue plays a defined role in catalysis: the serine acts as the nucleophile, the histidine functions as a general base, and the aspartate stabilizes the protonation state of the histidine. Together, these three residues catalyze ester bond hydrolysis (Rauwerdink and Kazlauskas, 2015). The prototypical PETase, from *P. sakaiensis* (PDB ID: pdb_00005xjh (Joo et al., 2018)), contains such a canonical catalytic triad in its catalytic center, supporting PET depolymerization into MHET and downstream products. Using this structure as a search template, three active site residues (Ser 148 [auth 160], Asp 194 [auth 206], and His 225 [auth 237], Supplementary Table S1) can be directly selected in the Mol* 3D viewer (Figure 1H) or chosen from the residue dropdown (Figure 1I), providing the 3D motif query that captures the spatial arrangement of catalytic residues. (N.B.: [auth ###] refers to an author-designated residue number, which can differ from the UniProt residue number.) Advanced Options include RMSD Cutoff and Atom Pairing (Figure 1J). The RMSD Cutoff is used to filter results by the RMSD between the query motif and each hit, with the default cutoff being 2 Å. Atom Pairing options include All Atoms, Side-Chain Atoms, Backbone Atoms, or Alpha & Beta Carbon. This Atom Pairing setting controls which atom sets are used to compute RMSD.

Combined searching using Structure Attribute, Sequence Motif, and 3D Motif Search tools returned 126 structures as of August 2026 (Supplementary Table S2). Predictably, most of these structures are bacterial proteins (104 structures), including many from *P. sakaiensis* (38 structures). Search results also include 18 structures from eukaryotes, including structures from fungi and humans, together with two structures from archaea. Of these 126 structures, 75 are structures of verified PET-degrading enzymes based on characterization in the literature and annotations in PlasticDB and PaZy (Gambarini et al., 2022; Buchholz et al., 2022). Both fungi and archaea are known to have species that perform PET degradation via serine hydrolase enzymes, whereas no human enzymes are known to degrade PET via a serine hydrolase mechanism, despite some of them exhibiting amino acid sequence and 3D structural features found in PET-degrading enzymes (Buchholz et al., 2022; Gambarini et al., 2022). A few human enzymes have been shown to degrade PET via alternative mechanisms (Huang et al., 2024), but these enzymes do not rely upon the catalytic triad and, therefore, would be detected by this search.

Search results can be displayed as Structures, Macromolecules, Assemblies, or Ligands, depending on which output suits the user’s needs. Selecting Structure view shows individual PDB entries or CSMs, each corresponding to a single deposition or predicted model. The Macromolecules view shows polymeric sequences present in structures, whereas the Assemblies view presents relevant biological assemblies. The Ligand view lists all non-polymeric chemical components found in structures. If the user wants to only view unique structures (i.e., to view only one PsPETase structure and one LCC structure although multiple structures of each are available in the PDB), users may utilize the Grouping options in the Macromolecule view (e.g., Group Results by Sequence Identity or by UniProt Accession) (Burley et al., 2025).

By combining these different types of searches, RCSB.org users can efficiently retrieve and explore both experimentally determined structures and CSMs relevant to PET hydrolysis, facilitating comparative analyses and hypothesis generation for enzyme function and engineering.

### 3.2 Visualizing PET-degrading enzymes with sequence annotations in 3D

The RCSB.org Sequence Annotations in 3D tool (Segura et al., 2022) integrates the RCSB PDB Sequence Annotations Viewer (Figure 2A) (Segura et al., 2020) with Mol* (Sehnal et al., 2021) to provide synchronized, interactive visualization of positional features in protein and nucleic acid sequences alongside their corresponding 3D biostructures. Positional features are aggregated from multiple trusted bioinformatics resources, including annotations derived from PDB entries themselves, UniProt and RefSeq records (Figure 2B), membrane protein databases, and domain classification resources (Figure 2C). Annotations are organized within distinct horizontal tracks encompassing a wide range of biochemical and structural properties, including residue geometry measurements, secondary structure features (Figure 2D), sequence variants and mutations (Figure 2E), post-translational modifications, catalytic or active sites (Figure 2F), and intermolecular interfaces (Figure 2G). Users can interactively select and highlight positional features to visualize their spatial localization within both the sequence (1D) and structure (3D), enabling identification of interactions and spatial patterns that may not be apparent from analysis of linear polymer sequences alone.

**FIGURE 2:**
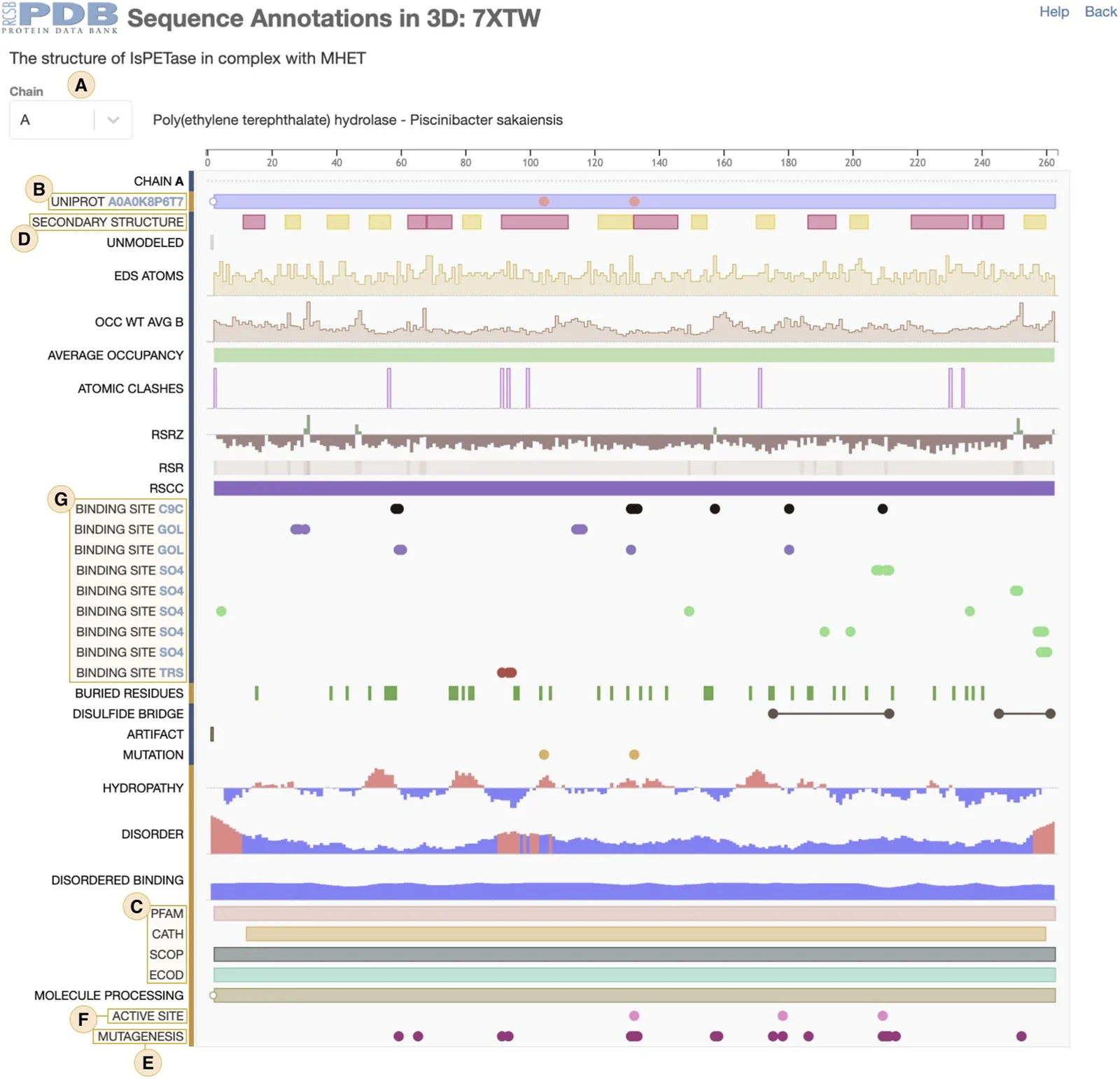
Sequence Annotations show positional features of protein sequences. **(A)** Sequence Display Panel shows the positional features of the protein sequence. **(B)** UniProt records of the protein sequence. **(C)** Domain Classification resources including PFAM, CATH, SCOP, and ECOD. **(D)** Secondary Structure records. **(E)** Residues for which site-directed mutagenesis has been performed in corresponding PDB structures. **(F)** Active site residues. **(G)** Interface residues for ligand binding sites. (N.B.: wwPDB is transitioning the PDB ID format from four characters to 12 (Feng et al., 2026). In the future, PDB ID 7xtw will be re-identified as pdb_00007xtw.).

For PsPETase, the Sequence Annotations in 3D tool enables visualization of positional features governing PET hydrolase activity. Since substrate binding sites are crucial for understanding enzymatic activity, a ligand-bound structure (PDB ID pdb_00007xtw (Yang et al., 2023)) proves most insightful for visualization and analysis. Alongside the Sequence Display Panel (Figure 2), Structure Display Panel uses the Mol* application to show the amino acid positions of the PsPETase on a 3D canvas (Figure 3A). The UniProt sequence is aligned with the protein sequence, conveniently highlighting mismatches in red, such that mutations can be easily identified (Figure 3B). In this MHET-bound PsPETase structure (PDB ID pdb_00007xtw), the catalytic Ser is mutated to Ala to prevent cleavage of the MHET ester bond (Yang et al., 2023). Selection of positional features in the sequence, such as active site residues, triggers the same selection in the Mol* viewer (Figure 3C). Once the catalytic triad residues (Ala132 [auth 160] (natively Ser), Asp178 [auth 206], and His209 [auth 237], Supplementary Table S1) are selected in Mol*, they can be visualized in ball-and-stick representation to view their proximity to the MHET substrate (Figure 3D). The carbonyl group of the MHET ester is situated near Ala132, which would support nucleophilic attack by serine in the wild-type enzyme.

**FIGURE 3:**
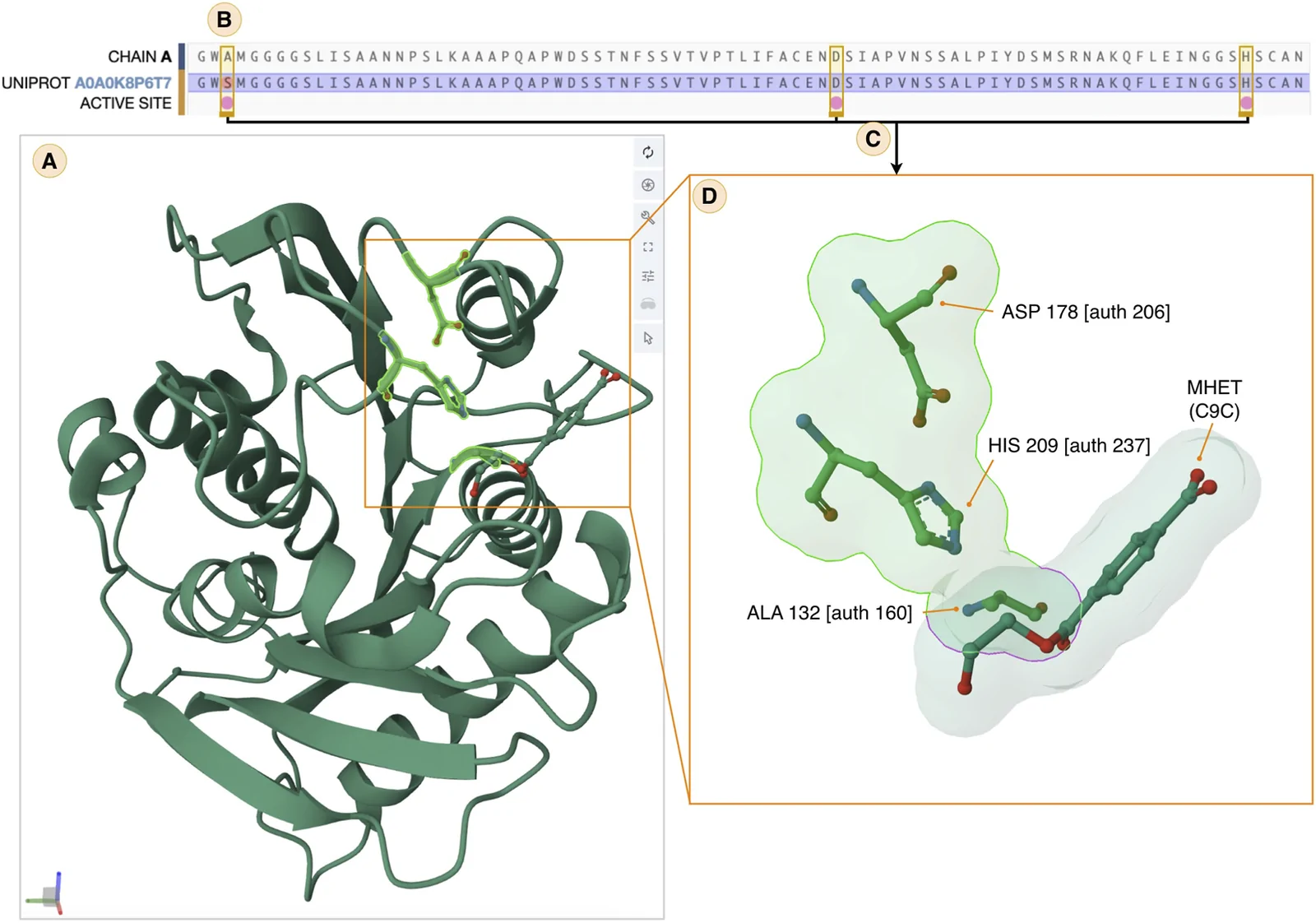
Sequence Annotation in 3D enables selection and visualization of active site residues. **(A)** Structure Display Panel uses the Mol* application to show the Cartesian coordinates of the protein on a 3D canvas. **(B)** Mismatches between the protein sequence and UniProt are highlighted in red. **(C)** Sequence and Structure Display Panels are dynamically connected so selecting/deselecting a region or residue in one of them will trigger the same selection/deselection in the other. **(D)** Visualization of MHET ligand and active site residues Ala132 [auth 160] (wild-type Ser), Asp178 [auth 206], and His209 [auth 237] in PsPETase.

For PETases, the PET degradation product MHET is hypothesized to mimic the substrate binding mode because the MHET monomer harbors the same chemical moieties as the PET polymer, such that the same moieties drive binding to PETase (Yang et al., 2023). Using binding site annotations, amino acid residues involved in MHET (Chemical Component Dictionary or CCD ID C9C) binding can be identified, selected, and visualized (Figure 4). These residues form a narrow binding cleft, stabilizing the binding of MHET (Figure 4A). This invagination is lined primarily with hydrophobic and aromatic residues, including: Gly58 [auth 86], Tyr59 [auth 87], Trp131 [auth 159], Ala132 [auth 160], Met133 [auth 161], Trp157 [auth 185], Ile180 [auth 208], and His209 [auth 237] (Figure 4B; Supplementary Table S1). Backbone amide N–H groups of Tyr59 and Met133 collectively form the oxyanion hole (Figure 4C), which stabilizes the oxyanion formed upon nucleophilic attack of the ester carbonyl.

**FIGURE 4:**
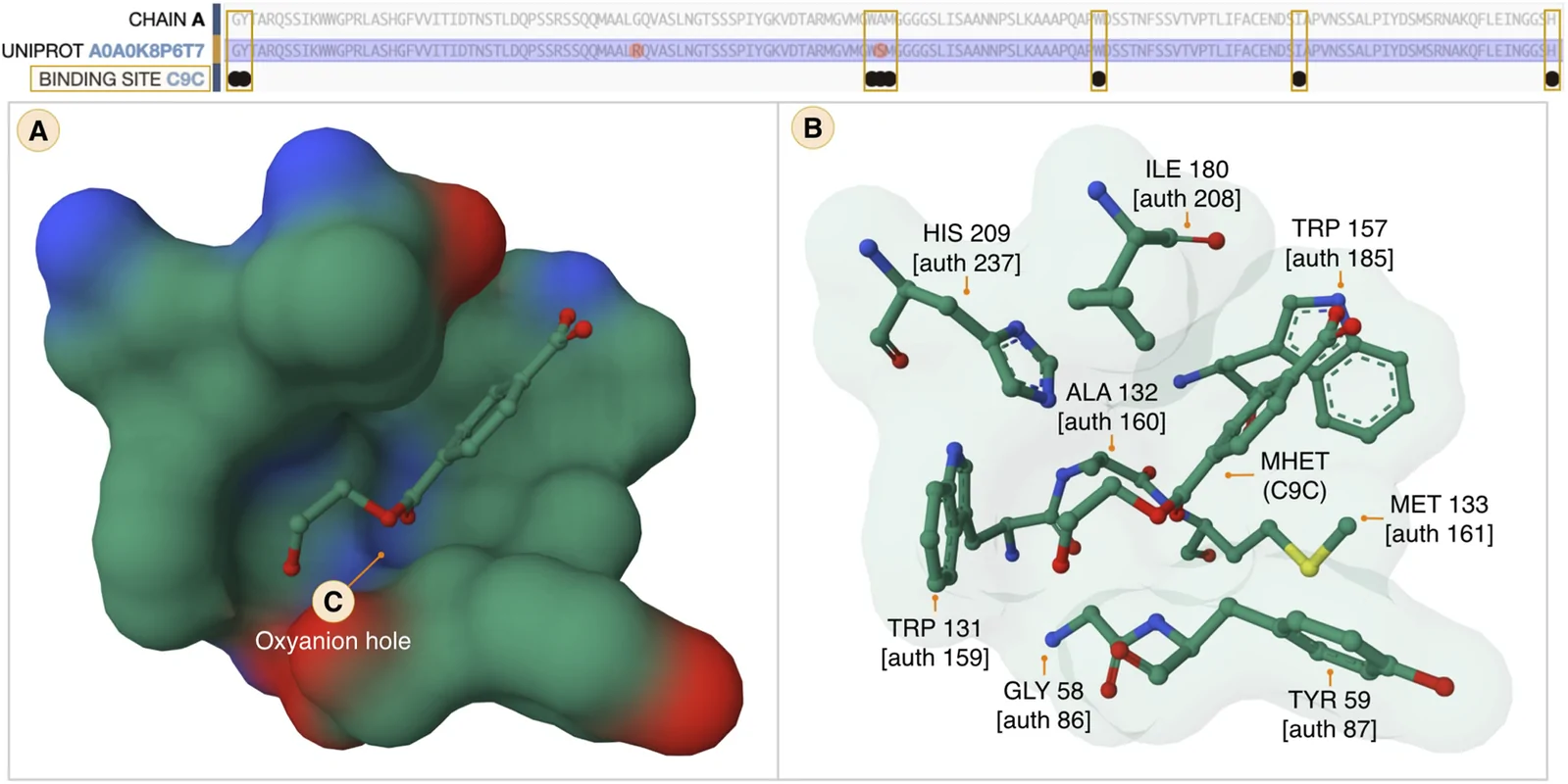
Sequence Annotation in 3D enables selection and visualization of ligand binding residues. **(A)** Molecular Surface Representation of ligand binding residues provides a view of the MHET binding cleft. **(B)** Ball-and-Stick Representation of ligand binding residues provides a view of individual residues within the MHET binding cleft. **(C)** Coloring the surface by Element Symbol provides a clear representation of the oxyanion hole, made up of backbone amide N–H groups, colored in blue.

Our Sequence Annotation in 3D tool supports molecular graphics displays for facile identification of key residues, such as active-site and/or ligand-binding residues. Integration of sequence selection with structure selection at RCSB.org enables visualization of these key residues with diverse representation options, including ball-and-stick and surface representations via the Mol* interface. Dynamic connections between 1D sequence and 3D structure analyses allow users to generate insightful visualizations for understanding enzymatic structure-function relationships. Deepening understanding of key functional residues is essential for enzyme engineering.

### 3.3 Analyzing PET-degrading enzymes via pairwise structure alignment

To demonstrate the utility of the Pairwise Structure Alignment tool (Bittrich et al., 2024), we applied this tool to PsPETase structures (Figure 5), comparing the wild type/apo structure (PDB ID: pdb_00005xg0 (Han et al., 2017)), a substrate analog-bound structure (PDB ID: pdb_00007xtw), and a predicted CSM (CSM ID: AF_AFA0A0K8P6T7F1). Superposition of these structures reveals local conformational differences associated with ligand binding, most notably a rearrangement of a tryptophan residue in the active site implicated in substrate accommodation (Figure 5A; Supplementary Table S1). Despite these local differences, the aligned structures exhibit low RMSD values, indicating strong global structural similarity across apo, ligand-bound, and predicted structures (Figure 5B). The predicted CSM is structurally similar to both experimentally determined structures (Figure 5C), particularly within the catalytic center, supporting the reliability of computational predictions for this enzyme family. PsPETase is currently regarded as the most efficient PET-degrading enzyme at ambient temperatures, and 3D structural comparisons across apo, ligand-bound, and predicted CSMs provide mechanistic insight into catalytic function.

**FIGURE 5:**
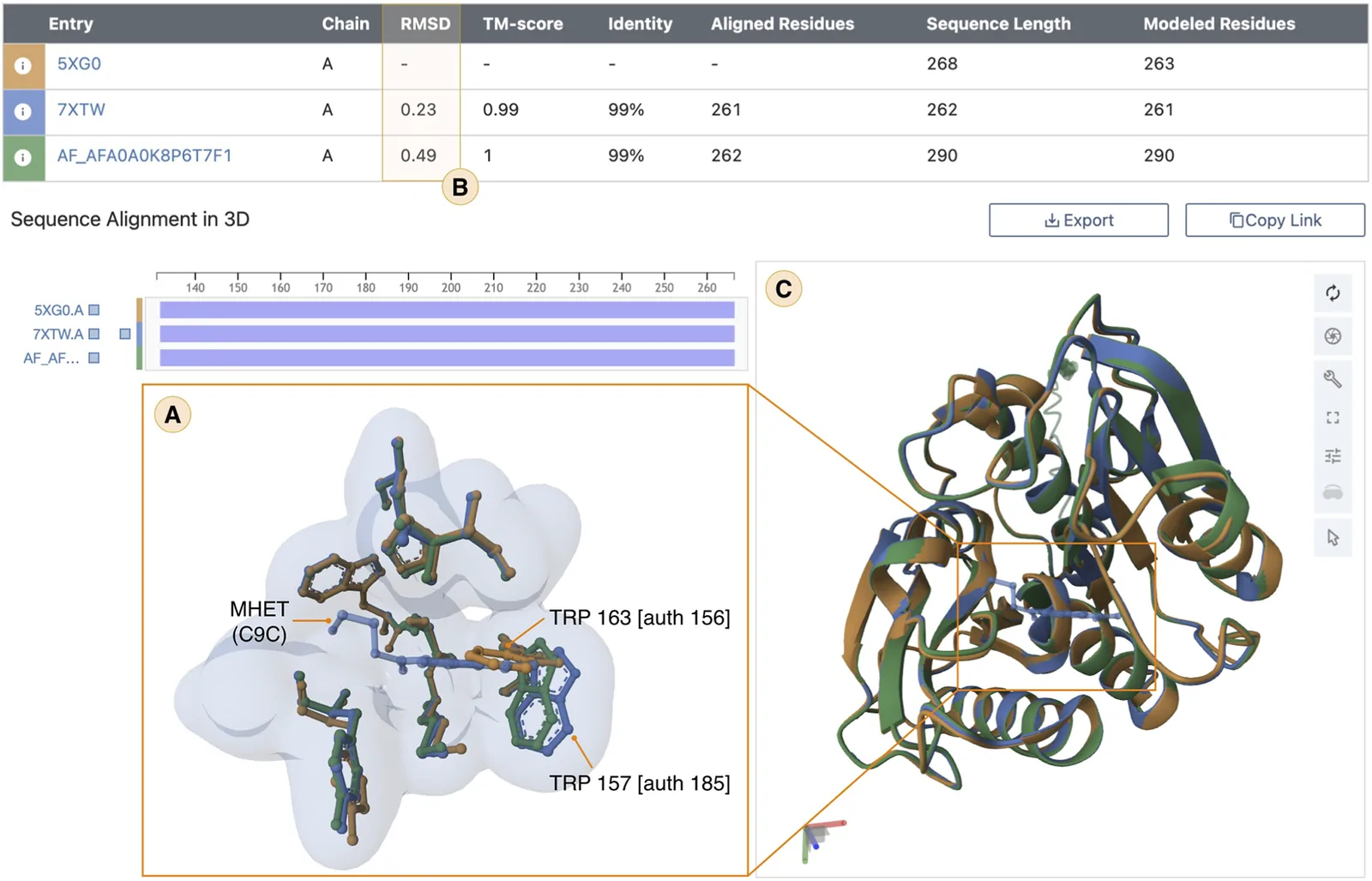
Structural comparison of PsPETases: wild type/apo (pdb_00005xg0, orange), S160A variant with substrate analog-bound (pdb_00007xtw, blue), and predicted CSM (AF_AFA0A0K8P6T7F1, green) generated using the RCSB PDB Pairwise Structure Alignment tool. **(A)** Superposition highlighting localized conformational changes upon ligand binding, including rearrangement of an active-site tryptophan residue implicated in substrate accommodation (Trp163 [auth 156] in pdb_00005xg0 chain A and Trp157 [auth 185] in pdb_00007xtw chain A). **(B)** Global structural alignment demonstrating sub-Ångstrom unit RMSD values between apo, ligand-bound, and predicted forms, indicative of overall structural conservation. **(C)** Overlay of the predicted CSM with experimentally determined structures, showing high structural agreement within the catalytic core.

Extending this analysis to enzymes with different temperature optima further highlights the value of 1D and 3D structural alignments. Comparative superposition of PsPETase (PDB ID: pdb_00007xtw) and Leaf-branch compost cutinase (LCC) (PDB ID: pdb_00007vve (Zeng et al., 2022)) (Figure 6) reveals structural determinants associated with temperature-dependent differences in catalytic activity. PsPETase displays greater active site flexibility, evidenced by displacement of the active site tryptophan (Trp157 [auth 185] in PsPETase and Trp157 [auth 190] in LCC) (Figure 6A; Supplementary Table S1). Movement of this Trp sidechain creates more space in the active site, allowing the MHET substrate analog to sit deeper in the active site groove of the enzyme (Figure 6B). Increased flexibility is attributable to amino acid substitutions that enhance catalytic efficiency at ambient temperatures, e.g., serine (Ser186 [auth 214]) in PsPETase and histidine (His185 [auth 218]) in LCC (Figure 6C; Supplementary Table S1), and isoleucine (Ile190 [auth 218]) in PsPETase and phenylalanine (Phe189 [auth 222]) in LCC (Figure 6D; Supplementary Table S1). In contrast, LCC exhibits a more rigid architecture, potentially consistent with increased thermostability and superior performance at elevated temperatures. Visualization of these structural differences provides insights into the distinct catalytic profiles of the two enzymes at the atomic level.

**FIGURE 6:**
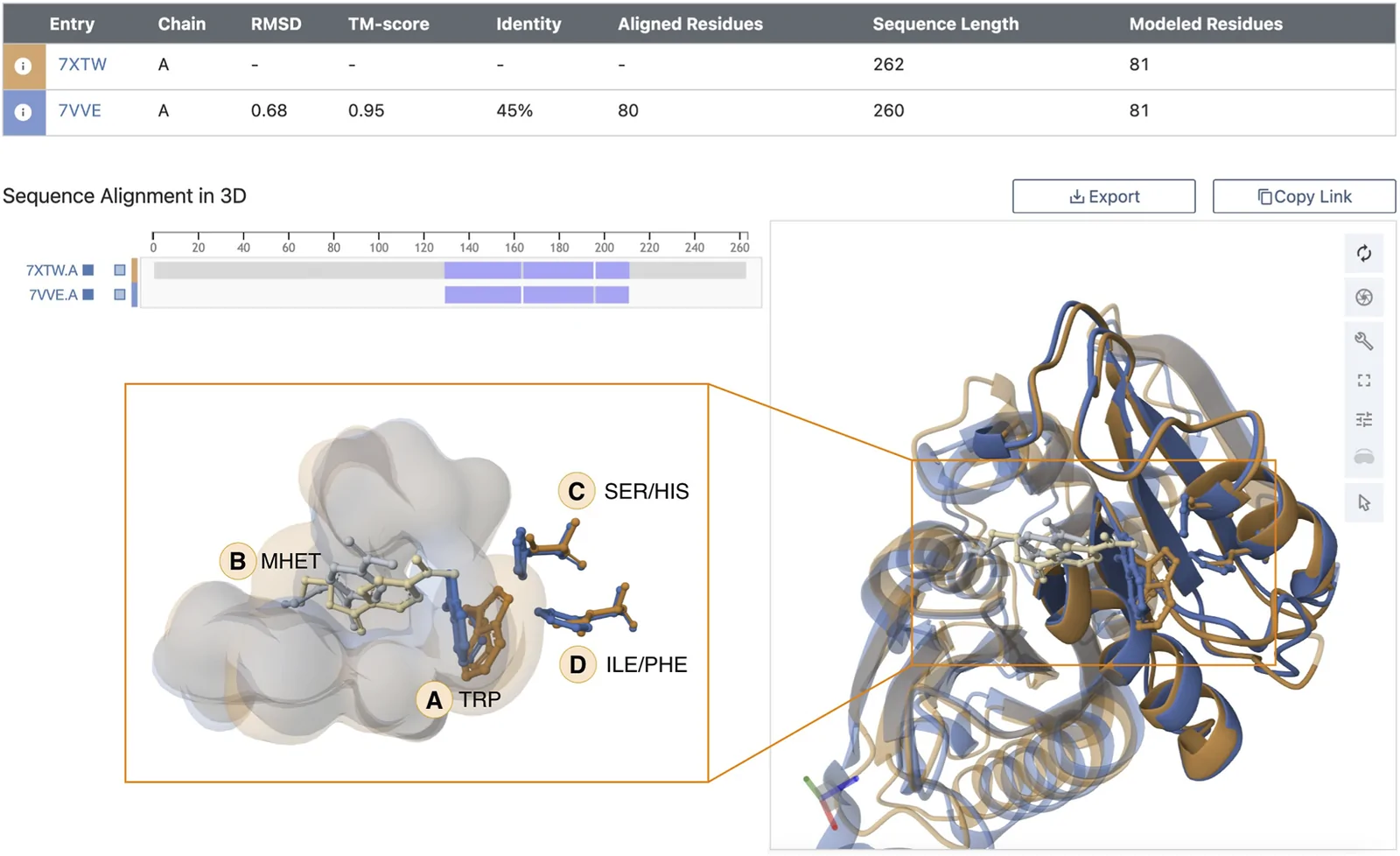
Structural comparison of PsPETase (pdb_00007xtw, orange) and Leaf-branch compost cutinase (pdb_00007vve, blue) generated using the RCSB PDB Pairwise Structure Alignment tool. The alignment reveals displacement of the active site tryptophan (Trp157 [auth 185] in PsPETase and Trp157 [auth 190] in LCC) **(A)** and the MHET ligand **(B)**. Active site flexibility in PsPETase is afforded by amino acid substitutions, e.g., Ser186 [auth 214] in PsPETase and His185 [auth 218] in LCC **(C)** plus Ile190 [auth 218] in PsPETase and Phe189 [auth 222] in LCC **(D)**.

Pairwise 3D structure alignment also facilitates the analysis of engineered variants. For example, comparison of LCC wild type (PDB ID: pdb_00004eb0 (Sulaiman et al., 2014)) and an engineered LCC variant (PDB ID: pdb_00006tht (Tournier et al., 2020)) (Figure 7) allows visualization of amino acid substitutions that improve thermostability and catalytic efficiency. Superposition of the two structures in 3D highlights five single amino acid substitutions (i.e., Tyr127Gly, Ser165Ala, Phe243Ile, Asp238Cys, and Ser283Cys, Supplementary Table S1), revealing the structural consequences thereof within the α/β-hydrolase fold. For structural characterization of the engineered LCC, a catalytically inactive variant was studied, wherein the catalytic Ser165 is mutated to Ala, because this variant produced higher-quality crystals (Tournier et al., 2020). Tyr127 and Phe243 flank the substrate binding cleft (Figure 7A). The Phe243Ile substitution supports improved enzyme activity by expanding access to the substrate binding site, whereas the Tyr127Gly substitution increases the melting temperature of LCC, a measure of improved thermostability. Moreover, the engineered Asp238Cys and Ser283Cys substitutions form a disulfide bond (Figure 7B) that contributes to thermal stabilization by reinforcing local structural rigidity. Together, these modifications reinforce stabilizing interactions and reduce conformational flexibility at high temperatures. Such analyses underpin rational protein engineering strategies, including industrial optimization efforts aimed at enhancing LCC performance for higher-temperature PET degradation.

**FIGURE 7:**
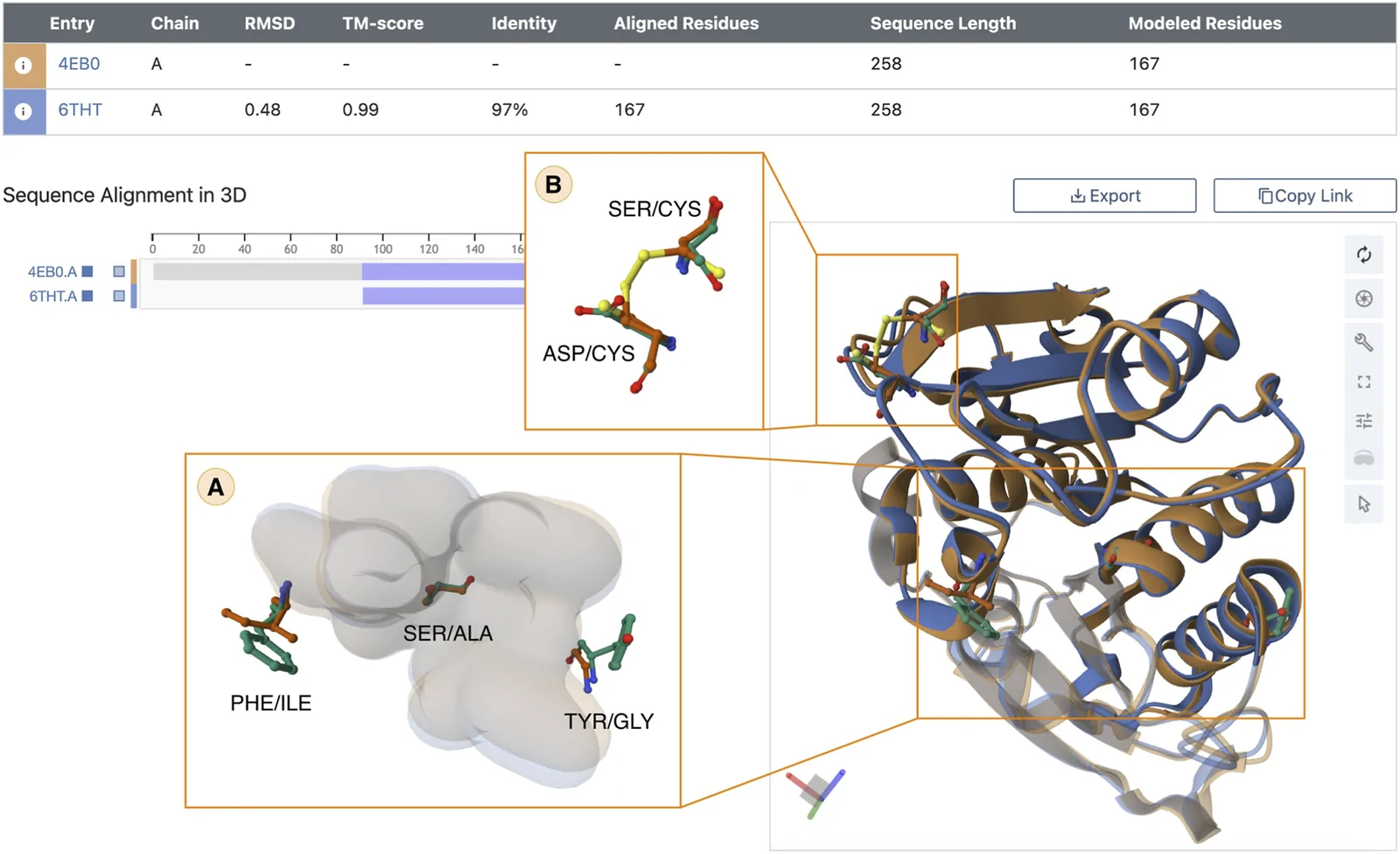
Pairwise Structure Alignment of Leaf-branch compost cutinases: wild type (pdb_00004eb0, orange) and engineered for improved thermostability (pdb_00006tht, blue). **(A)** Mutation of S165 of the catalytic triad renders the enzyme variant inactive. The Tyr127Gly and Phe243Ile substitutions lie at substrate binding site-flanking positions. **(B)** The disulfide formed by the Asp238Cys and Ser283Cys residue pair contributes to thermal stabilization.

Overall, the Pairwise Structure Alignment tool provides a comprehensive framework for integrating 3D structural comparisons, functional interpretation, and protein engineering analysis. By enabling direct visualization of conformational variability, substrate-induced rearrangements, and engineered modifications, this tool supports better mechanistic understanding of PET hydrolysis and facilitates structure-guided optimization of PET-degrading enzymes.

## 4 Discussion

Given how rapidly plastic generation outpaces plastic recycling, improved tools for managing plastic waste are critically important in the modern era. Plastic-degrading enzymes can serve as a tool for plastic breakdown, after which plastic monomers can be valorized or recycled. To effectively use plastic-degrading enzymes, such as PETase and LCC, at industrial scale, a deep basic understanding of these enzymes is required to drive engineering efforts.

RCSB.org supports exploration of public domain 3D biostructures and provides training materials via PDB-101 (https://PDB101.rcsb.org (Zardecki et al., 2022)) to help researchers and students alike utilize PDB data. PDB-101 training materials include Molecule of the Month articles that explain the structure and function of important molecules [e.g., ref], and webinars, which make biotechnology topics and research tools accessible to a wide range of researchers from beginner to expert (e.g., Workhorses of Biotechnology). For structure exploration, RCSB.org provides Advanced Search tools including Structure Attribute Search, Sequence Motif Search, and 3D Motif Search that offer robust selection of search criteria allowing construction of powerful searches, and the option to include CSMs vastly expands available search space. RCSB.org Advanced Search additionally supports full Boolean logic, enabling flexible, modular searches.

Beyond identifying 3D biostructures of interest, RCSB.org supports structure visualization and analysis. PDB structures can be visualized using the Sequence Annotations in 3D tool, which highlights important features including sequence mutations, active site residues, and ligand binding sites. Integration with Mol* allows facile selection of functionally important residues in the structure, which in turn facilitates 3D rendering of key structural snapshots for functional understanding. For further structural analyses, Pairwise Structure Alignment enables insightful comparisons between related structures. In the case of PET-degrading enzymes, comparing the apo enzyme with the ligand-bound form demonstrates localized conformational changes that occur on ligand binding. Comparisons between PET-degrading enzymes from different species, such as PsPETase and LCC, reveal amino acid substitutions that underpin mechanistic differences, including catalytic efficiency at ambient temperatures and relative thermostability.

With the benefit of the RCSB PDB tools described herein, researchers can perform analyses necessary for rational protein design. Effective enzyme engineering depends critically on understanding structure-function relationships at the atomic level, which can be exploited to enable industrial use of enzymes in plastic biodegradation. Future efforts aimed at engineering plastic-degrading enzymes will almost certainly rely upon structural understanding of these enzymes in 3D at the atomic level and identification of amino acid substitutions that can contribute to enhanced catalytic activity and superior thermostability.

## Data Availability

The original contributions presented in the study are included in the article/Supplementary Material. Resources described are freely available at RCSB.org and GitHub repositories: github.com/rcsb/rcsb-saguaro-3d and github.com/rcsb/rcsb-pecos-app. Further inquiries can be directed to the corresponding author.

## References

[B1] Baek M. DiMaio F. Anishchenko I. Dauparas J. Ovchinnikov S. Lee G. R. (2021). Accurate prediction of protein structures and interactions using a three-track neural network. Science 373, 871–876. 10.1126/science.abj8754 PMC7612213 34282049

[B2] Basner J. (2017). “Impact Analysis of Berman HM et al,” in The Protein Data Bank.

[B3] Bell E. L. Smithson R. Kilbride S. Foster J. Hardy F. J. Ramachandran S. (2022). Directed evolution of an efficient and thermostable PET depolymerase. Nat. Catal. 5, 673–681. 10.1038/s41929-022-00821-3

[B4] Berman H. M. Westbrook J. Feng Z. Gilliland G. Bhat T. N. Weissig H. (2000). The protein data bank. Nucleic Acids Res. 28, 235–242. 10.1093/nar/28.1.235 10592235 PMC102472

[B5] Berman H. M. Henrick K. Nakamura H. (2003). Announcing the worldwide protein data bank. Nat. Struct. Biol. 10, 980. 10.1038/nsb1203-980 14634627

[B6] Bittrich S. Segura J. Duarte J. M. Burley S. K. Rose Y. (2024). RCSB protein data bank: exploring protein 3D similarities via comprehensive structural alignments. Bioinformatics 40, btae370. 10.1093/bioinformatics/btae370 PMC11212067 38870521

[B7] Brackmann R. de Oliveira Veloso C. de Castro A. M. Langone M. A. P. (2023). Enzymatic post-consumer poly(ethylene terephthalate) (PET) depolymerization using commercial enzymes. 3 Biotech. 13, 135. 10.1007/s13205-023-03555-6 PMC10130296 37124991

[B8] Buchholz P. C. F. Feuerriegel G. Zhang H. Perez-Garcia P. Nover L.-L. Chow J. (2022). Plastics degradation by hydrolytic enzymes: the plastics-active enzymes database—PAZy. Proteins Struct. Funct. Bioinf 90, 1443–1456. 10.1002/prot.26325 35175626

[B9] Burley S. K. Bhikadiya C. Bi C. Bittrich S. Chen L. Crichlow G. V. (2022). RCSB protein data bank: celebrating 50 years of the PDB with new tools for understanding and visualizing biological macromolecules in 3D. Protein Sci. 31, 187–208. 10.1002/pro.4213 34676613 PMC8740825

[B10] Burley S. K. Bhikadiya C. Bi C. Bittrich S. Chao H. Chen L. (2023). RCSB protein data bank (RCSB.org): delivery of experimentally-determined PDB structures alongside one million computed structure models of proteins from artificial intelligence/machine learning. Nucleic Acids Res. 51, D488–D508. 10.1093/nar/gkac1077 36420884 PMC9825554

[B11] Burley S. K. Bhatt R. Bhikadiya C. Bi C. Biester A. Biswas P. (2025). Updated resources for exploring experimental PDB structures and computed structure models at the RCSB protein data bank. Nucleic Acids Res. 53, D564–D574. 10.1093/nar/gkae1091 39607707 PMC11701563

[B12] Carniel A. Valoni É. Nicomedes J. Gomes A. d. C. Castro A. M. d. (2017). Lipase from *Candida antarctica* (CALB) and cutinase from *humicola Insolens* act synergistically for PET hydrolysis to terephthalic acid. Process Biochem. 59, 84–90. 10.1016/j.procbio.2016.07.023

[B13] Chen Y. Lin Z. Yang S. (1998). Plasticization and crystallization of poly(ethylene terephthalate) induced by water. J. Therm. Anal. Calorim. 52, 565–568. 10.1023/a:1010123723719

[B14] Cui Y. Chen Y. Liu X. Dong S. Tian Y. e. Qiao Y. (2021). Computational redesign of a PETase for plastic biodegradation under ambient condition by the GRAPE strategy. ACS Catal. 11, 1340–1350. 10.1021/acscatal.0c05126

[B15] Feng Z. Verdiguel N. Di Costanzo L. Goodsell D. S. Westbrook J. D. Burley S. K. (2020). Impact of the protein data bank across scientific disciplines. Data Sci. J. 19, 1–14. 10.5334/dsj-2020-025

[B16] Feng Z. Balasubramaniyan B. Bekker G.-J. Duarte J. M. Guranovic V. Henry J. (2026). Protein data bank (PDB) archive: a new architecture (beta) for scalable, PDBx/mmCIF-based data distribution. Acta Crystallogr. D. Struct. Biol. 82, 982–989. 10.1107/S2059798326006194 42467887 PMC13434931

[B17] Gambarini V. Pantos O. Kingsbury J. M. Weaver L. Handley K. M. Lear G. (2022). PlasticDB: a database of microorganisms and proteins linked to plastic biodegradation. Database (Oxford) 2022, baac008. 10.1093/database/baac008 35266524 PMC9216477

[B18] Geyer R. Jambeck J. R. Law K. L. (2017). Production, use, and fate of all plastics ever made. Sci. Adv. 3, e1700782. 10.1126/sciadv.1700782 28776036 PMC5517107

[B19] Han X. Liu W. Huang J.-W. Ma J. Zheng Y. Ko T.-P. (2017). Structural insight into catalytic mechanism of PET hydrolase. Nat. Commun. 8, 2106. 10.1038/s41467-017-02255-z 29235460 PMC5727383

[B20] Hu X. Thumarat U. Zhang X. Tang M. Kawai F. (2010). Diversity of polyester-degrading bacteria in compost and molecular analysis of a thermoactive esterase from *Thermobifida alba* AHK119. Appl. Microbiol. Biotechnol. 87, 771–779. 10.1007/s00253-010-2555-x 20393707

[B21] Huang X. Li Y. Shu Z. Huang L. Liu Q. Jiang G. (2024). High-efficiency degradation of PET plastics by glutathione S-Transferase under mild conditions. Environ. Sci. Technol. 58, 13358–13369. 10.1021/acs.est.4c02132 39012182

[B22] Joo S. Cho I. J. Seo H. Son H. F. Sagong H.-Y. Shin T. J. (2018). Structural insight into molecular mechanism of poly(ethylene terephthalate) degradation. Nat. Commun. 9, 382. 10.1038/s41467-018-02881-1 29374183 PMC5785972

[B23] Kenyon K. W. Kridler E. (1969). Laysan albatrosses swallow indigestible matter. Auk 86, 339–343. 10.2307/4083505

[B24] Kim H. R. Song W. S. (2006). Lipase treatment of polyester fabrics. Fibers Polym. 7, 339–343. 10.1007/bf02875764

[B25] Lafita A. Bliven S. Prlic A. Guzenko D. Rose P. W. Bradley A. (2019). BioJava 5: a community driven open-source bioinformatics library. PLoS Comput. Biol. 15, e1006791. 10.1371/journal.pcbi.1006791 30735498 PMC6383946

[B26] Lord C. C. Thomas G. Brown J. M. (2013). Mammalian alpha beta hydrolase domain (ABHD) proteins: lipid metabolizing enzymes at the interface of cell signaling and energy metabolism. Biochim. Biophys. Acta Mol. Cell Biol. Lipids 1831, 792–802. 10.1016/j.bbalip.2013.01.002 23328280 PMC4765316

[B27] Lu H. Diaz D. J. Czarnecki N. J. Zhu C. Kim W. Shroff R. (2022). Machine learning-aided engineering of hydrolases for PET depolymerization. Nature 604, 662–667. 10.1038/s41586-022-04599-z 35478237

[B28] Ma J. Wang S. (2014). “Chapter five - algorithms, applications, and challenges of protein structure alignment,” in Advances in Protein Chemistry and Structural Biology. Editor DONEV R. (Academic Press). 10.1016/B978-0-12-800168-4.00005-6 24629187

[B29] Markosian C. Di Costanzo L. Sekharan M. Shao C. Burley S. K. Zardecki C. (2018). Analysis of impact metrics for the protein data bank. Sci. Data 5, 180212. 10.1038/sdata.2018.212 30325351 PMC6190746

[B30] Marten E. Müller R.-J. Deckwer W.-D. (2005). Studies on the enzymatic hydrolysis of polyesters. II. Aliphatic–aromatic copolyesters. Polym. Degrad. Stab. 88, 371–381. 10.1016/j.polymdegradstab.2004.12.001

[B31] Müller R.-J. Schrader H. Profe J. Dresler K. Deckwer W.-D. (2005). Enzymatic degradation of poly(ethylene terephthalate): rapid hydrolyse using a hydrolase from *T. fusca* . Macromol. Rapid Commun. 26, 1400–1405. 10.1002/marc.200500410

[B32] Nicholson J. L. Leighton G. R. (1942). Plastics Come of Age. Harper’s Magazine, 306.

[B33] Piehl D. W. Vallat B. Truong I. Morsy H. Bhatt R. Blaumann S. (2025). rcsb-api: python toolkit for streamlining access to RCSB protein data bank APIs. J. Mol. Biol. 437, 168970. 10.1016/j.jmb.2025.168970 39894387 PMC13137918

[B34] Rauwerdink A. Kazlauskas R. J. (2015). How the same core catalytic machinery catalyzes 17 different reactions: the serine-histidine-aspartate catalytic triad of α/β-hydrolase fold enzymes. ACS Catal. 5, 6153–6176. 10.1021/acscatal.5b01539 28580193 PMC5455348

[B35] Ribitsch D. Heumann S. Trotscha E. Herrero Acero E. Greimel K. Leber R. (2011). Hydrolysis of polyethyleneterephthalate by *p*-nitrobenzylesterase from *Bacillus subtilis* . Biotechnol. Prog. 27, 951–960. 10.1002/btpr.610 21574267

[B36] Ribitsch D. Herrero Acero E. Greimel K. Dellacher A. Zitzenbacher S. Marold A. (2012). A new esterase from *Thermobifida halotolerans* hydrolyses polyethylene terephthalate (PET) and polylactic acid (PLA). Polymers 4, 617–629. 10.3390/polym4010617

[B37] Rillig M. C. (2012). Microplastic in terrestrial ecosystems and the soil? Environ. Sci. Technol. 46, 6453–6454. 10.1021/es302011r 22676039

[B38] Ritchie H. Roser M. (2018). Plastic pollution. Available online at: https://ourworldindata.org/plastic-pollution (Accessed August 25, 2026).

[B39] Rose Y. Duarte J. M. Lowe R. Segura J. Bi C. Bhikadiya C. (2021). RCSB protein data bank: architectural advances towards integrated searching and efficient access to macromolecular structure data from the PDB archive. J. Mol. Biol. 433, 166704. 10.1016/j.jmb.2020.11.003 33186584 PMC9093041

[B40] Rose Y. Brown R. Voigt M. Bhikadiya C. Bittrich S. Duarte J. M. (2026). RCSB protein data bank: next-Generation advanced search for exploration of experimental structures and computed structure models. Protein Sci. 35, e70731. 10.1002/pro.70731 42517697 PMC13410952

[B41] Segura J. Rose Y. Westbrook J. Burley S. K. Duarte J. M. (2020). RCSB protein data bank 1D tools and services. Bioinformatics 36, 5526–5527. 10.1093/bioinformatics/btaa1012 PMC8016458 33313665

[B42] Segura J. Rose Y. Bittrich S. Burley S. K. Duarte J. M. (2022). RCSB protein data bank 1D3D module: displaying positional features on macromolecular assemblies. Bioinformatics 38, 3304–3305. 10.1093/bioinformatics/btac317 PMC9191206 35543462

[B43] Sehnal D. Bittrich S. Deshpande M. Svobodova R. Berka K. Bazgier V. (2021). Mol* viewer: modern web app for 3D visualization and analysis of large biomolecular structures. Nucleic Acids Res. 49, W431–W437. 10.1093/nar/gkab314 PMC8262734 33956157

[B44] Silva C. M. Carneiro F. O'Neill A. Fonseca L. P. Cabral J. S. M. Guebitz G. (2005). Cutinase—A new tool for biomodification of synthetic fibers. J. Polym. Sci. A Polym. Chem. 43, 2448–2450. 10.1002/pola.20684

[B45] Son H. F. Cho I. J. Joo S. Seo H. Sagong H.-Y. Choi S. Y. (2019). Rational protein engineering of thermo-stable PETase from *Ideonella sakaiensis* for highly efficient PET degradation. ACS Catal. 9, 3519–3526. 10.1021/acscatal.9b00568

[B46] Sulaiman S. Yamato S. Kanaya E. Kim J.-J. Koga Y. Takano K. (2012). Isolation of a novel cutinase homolog with polyethylene terephthalate-degrading activity from leaf-branch compost by using a metagenomic approach. Appl. Environ. Microbiol. 78, 1556–1562. 10.1128/AEM.06725-11 22194294 PMC3294458

[B47] Sulaiman S. You D.-J. Kanaya E. Koga Y. Kanaya S. (2014). Crystal structure and thermodynamic and kinetic stability of metagenome-derived LC-cutinase. Biochemistry 53, 1858–1869. 10.1021/bi401561p 24593046

[B48] Tarazona N. A. Wei R. Brott S. Pfaff L. Bornscheuer U. T. Lendlein A. (2022). Rapid depolymerization of poly(ethylene terephthalate) thin films by a dual-enzyme system and its impact on material properties. Chem. Catal. 2, 3573–3589. 10.1016/j.checat.2022.11.004 PMC10284027 37350932

[B49] Tauriello G. Waterhouse A. M. Haas J. Behringer D. Bienert S. Garello T. (2025). ModelArchive: a deposition database for computational macromolecular structural models. J. Mol. Biol. 437, 168996. 10.1016/j.jmb.2025.168996 PMC13370677 39947281

[B50] Thompson R. C. Olsen Y. Mitchell R. P. Davis A. Rowland S. J. John A. W. G. (2004). Lost at sea: where is all the plastic? Science 304, 838. 838-838. 10.1126/science.1094559 15131299

[B51] Tournier V. Topham C. M. Gilles A. David B. Folgoas C. Moya-Leclair E. (2020). An engineered PET depolymerase to break down and recycle plastic bottles. Nature 580, 216–219. 10.1038/s41586-020-2149-4 32269349

[B52] Tunyasuvunakool K. Adler J. Wu Z. Green T. Zielinski M. Zidek A. (2021). Highly accurate protein structure prediction for the human proteome. Nature 596, 590–596. 10.1038/s41586-021-03828-1 PMC8387240 34293799

[B53] van der Aalst W. M. P. Bichler M. Heinzl A. (2017). Responsible data science. Bus. Inf. Sys Eng. 59, 311–313. 10.1007/s12599-017-0487-z

[B54] Varadi M. Bertoni D. Magana P. Paramval U. Pidruchna I. Radhakrishnan M. (2024). AlphaFold protein structure database in 2024: providing structure coverage for over 214 million protein sequences. Nucleic Acids Res. 52, D368–D375. 10.1093/nar/gkad1011 PMC10767828 37933859

[B55] Wagner M. Scherer C. Alvarez-Muñoz D. Brennholt N. Bourrain X. Buchinger S. (2014). Microplastics in freshwater ecosystems: what we know and what we need to know. Environ. Sci. Eur. 26, 12. 10.1186/s12302-014-0012-7 28936382 PMC5566174

[B56] Wei R. Zimmermann W. (2017). Microbial enzymes for the recycling of recalcitrant petroleum-based plastics: how far are we? Microb. Biotechnol. 10, 1308–1322. 10.1111/1751-7915.12710 28371373 PMC5658625

[B57] Westbrook J. D. Young J. Y. Shao C. Feng Z. Guranovic V. Lawson C. (2022). PDBx/mmCIF ecosystem: foundational semantic tools for structural biology. J. Mol. Biol. 434, 167599. 10.1016/j.jmb.2022.167599 PMC10292674 35460671

[B64] wwPDB consortium (2019). Protein Data Bank: the single global archive for 3D macromolecular structure data. Nucleic Acids Res. 47, D520–D528. 10.1093/nar/gky949 PMC6324056 30357364

[B58] Wilkinson M. D. Dumontier M. Aalbersberg I. J. Appleton G. Axton M. Baak A. (2016). The FAIR guiding principles for scientific data management and stewardship. Sci. Data 3, 160018. 10.1038/sdata.2016.18 26978244 PMC4792175

[B59] Yang Y. Min J. Xue T. Jiang P. Liu X. Peng R. (2023). Complete bio-degradation of poly(butylene adipate-co-terephthalate) via engineered cutinases. Nat. Commun. 14, 1645. 10.1038/s41467-023-37374-3 PMC10039075 36964144

[B60] Yoshida S. Hiraga K. Takehana T. Taniguchi I. Yamaji H. Maeda Y. (2016). A bacterium that degrades and assimilates poly(ethylene terephthalate). Science 351, 1196–1199. 10.1126/science.aad6359 26965627

[B61] Zardecki C. Dutta S. Goodsell D. S. Lowe R. Voigt M. Burley S. K. (2022). PDB-101: educational resources supporting molecular explorations through biology and medicine. Protein Sci. 31, 129–140. 10.1002/pro.4200 PMC8740840 34601771

[B62] Zeng W. Li X. Yang Y. Min J. Huang J.-W. Liu W. (2022). Substrate-binding mode of a thermophilic PET hydrolase and engineering the enzyme to enhance the hydrolytic efficacy. ACS Catal. 12, 3033–3040. 10.1021/acscatal.1c05800 PMC9361285 35966606

[B63] Zhang Y. Skolnick J. (2005). TM-align: a protein structure alignment algorithm based on the TM-score. Nucleic Acids Res. 33, 2302–2309. 10.1093/nar/gki524 15849316 PMC1084323

