# Supplementary material for: RCSB PDB web portal RCSB.org for biotechnology research: exploration and visualization of plastic-degrading enzymes at the atomic level in 3D
Source: Front Bioeng Biotechnol. 2026 Oct 5;14:1947029. doi: 10.3389/fbioe.2026.1947029 (PMC13677904; doi:10.3389/fbioe.2026.1947029)
Supplement: Supplementary file 1 [file Supplementaryfile1.docx]

Supplementary Material

RCSB PDB Web Portal RCSB.org for Biotechnology Research: Exploration and Visualization of Plastic-Degrading Enzymes at the Atomic Level in 3D

Alison Biester, Christine Zardecki, Yana Rose, Joan Segura, Jose M. Duarte, Stephen K. Burley^*^

*** Correspondence:**
Stephen K. Burley
[Stephen.Burley@RCSB.org](mailto:Stephen.Burley@RCSB.org)

# 1 Supplementary Tables

**Table S1.** Numbering for all amino acid residues discussed in the manuscript (in the order presented in the text).

| Enzyme | PDB ID | Native Amino Acid | Amino Acid in Structure | Residue Number | Author Residue Number |
| --- | --- | --- | --- | --- | --- |
| *Ps*PETase | pdb_00005xjh | Serine | Serine | 148 | 160 |
| *Ps*PETase | pdb_00005xjh | Aspartate | Aspartate | 194 | 206 |
| *Ps*PETase | pdb_00005xjh | Histidine | Histidine | 225 | 237 |
| *Ps*PETase | pdb_00007xtw | Serine | Alanine | 132 | 160 |
| *Ps*PETase | pdb_00007xtw | Aspartate | Aspartate | 178 | 206 |
| *Ps*PETase | pdb_00007xtw | Histidine | Histidine | 209 | 237 |
| *Ps*PETase | pdb_00007xtw | Glycine | Glycine | 58 | 86 |
| *Ps*PETase | pdb_00007xtw | Tyrosine | Tyrosine | 59 | 87 |
| *Ps*PETase | pdb_00007xtw | Tryptophan | Tryptophan | 131 | 159 |
| *Ps*PETase | pdb_00007xtw | Methionine | Methionine | 133 | 161 |
| *Ps*PETase | pdb_00007xtw | Tryptophan | Tryptophan | 157 | 185 |
| *Ps*PETase | pdb_00007xtw | Isoleucine | Isoleucine | 180 | 208 |
| *Ps*PETase | pdb_00005xg0 | Tryptophan | Tryptophan | 163 | 156 |
| LCC | pdb_00007vve | Tryptophan | Tryptophan | 157 | 190 |
| *Ps*PETase | pdb_00007xtw | Serine | Serine | 186 | 214 |
| LCC | pdb_00007vve | Histidine | Histidine | 185 | 218 |
| *Ps*PETase | pdb_00007xtw | Isoleucine | Isoleucine | 190 | 218 |
| LCC | pdb_00007vve | Phenylalanine | Phenylalanine | 189 | 222 |
| LCC | pdb_0004eb0 | Tyrosine | Tyrosine | 92 | 127 |
| LCC | pdb_0006tht | Tyrosine | Glycine | 92 | 127 |
| LCC | pdb_0004eb0 | Serine | Serine | 130 | 165 |
| LCC | pdb_0006tht | Serine | Alanine | 130 | 165 |
| LCC | pdb_0004eb0 | Phenylalanine | Phenylalanine | 208 | 243 |
| LCC | pdb_0006tht | Phenylalanine | Isoleucine | 208 | 243 |
| LCC | pdb_0004eb0 | Aspartate | Aspartate | 203 | 238 |
| LCC | pdb_0006tht | Aspartate | Cysteine | 203 | 238 |
| LCC | pdb_0004eb0 | Serine | Serine | 248 | 283 |
| LCC | pdb_0006tht | Serine | Cysteine | 248 | 283 |

**Table S2.** Results of RCSB.org search shown in Figure 1 ([link](https://www.rcsb.org/search?request=%7B%22query%22%3A%7B%22type%22%3A%22group%22%2C%22logical_operator%22%3A%22and%22%2C%22nodes%22%3A%5B%7B%22type%22%3A%22group%22%2C%22logical_operator%22%3A%22and%22%2C%22nodes%22%3A%5B%7B%22type%22%3A%22group%22%2C%22nodes%22%3A%5B%7B%22type%22%3A%22terminal%22%2C%22service%22%3A%22text%22%2C%22parameters%22%3A%7B%22attribute%22%3A%22rcsb_polymer_entity.rcsb_ec_lineage.id%22%2C%22operator%22%3A%22in%22%2C%22negation%22%3Afalse%2C%22value%22%3A%5B%223.1.1.101%22%2C%223.1.1.1%22%2C%223.1.1.2%22%2C%223.1.1.3%22%2C%223.1.1.74%22%5D%7D%7D%5D%2C%22logical_operator%22%3A%22and%22%7D%5D%2C%22label%22%3A%22text%22%7D%2C%7B%22type%22%3A%22terminal%22%2C%22service%22%3A%22seqmotif%22%2C%22parameters%22%3A%7B%22value%22%3A%22GXSXG%22%2C%22pattern_type%22%3A%22simple%22%2C%22sequence_type%22%3A%22protein%22%7D%7D%2C%7B%22type%22%3A%22terminal%22%2C%22service%22%3A%22strucmotif%22%2C%22parameters%22%3A%7B%22value%22%3A%7B%22residue_ids%22%3A%5B%7B%22label_asym_id%22%3A%22A%22%2C%22struct_oper_id%22%3A%221%22%2C%22label_seq_id%22%3A148%7D%2C%7B%22label_asym_id%22%3A%22A%22%2C%22struct_oper_id%22%3A%221%22%2C%22label_seq_id%22%3A194%7D%2C%7B%22label_asym_id%22%3A%22A%22%2C%22struct_oper_id%22%3A%221%22%2C%22label_seq_id%22%3A225%7D%5D%2C%22entry_id%22%3A%225XJH%22%7D%2C%22rmsd_cutoff%22%3A2%2C%22atom_pairing_scheme%22%3A%22ALL%22%7D%7D%5D%7D%2C%22return_type%22%3A%22entry%22%2C%22request_options%22%3A%7B%22paginate%22%3A%7B%22start%22%3A0%2C%22rows%22%3A25%7D%2C%22results_content_type%22%3A%5B%22experimental%22%5D%2C%22scoring_strategy%22%3A%22combined%22%2C%22sort%22%3A%5B%7B%22sort_by%22%3A%22score%22%2C%22direction%22%3A%22desc%22%7D%5D%7D%2C%22request_info%22%3A%7B%22query_id%22%3A%2212492db1080030486224335662149e59%22%7D%7D)). Of 126 results, 75 are verified PETases.

| PDB ID | Verified PETase |
| --- | --- |
| pdb_00001aql | N |
| pdb_00001auo | N |
| pdb_00001aur | N |
| pdb_00001f6w | N |
| pdb_00001hlg | N |
| pdb_00001jmy | N |
| pdb_00001k8q | N |
| pdb_00001r1d | N |
| pdb_00001tqh | N |
| pdb_00001va4 | N |
| pdb_00002bce | N |
| pdb_00002fx5 | Y |
| pdb_00002h1i | N |
| pdb_00002veo | N |
| pdb_00003cn7 | N |
| pdb_00003cn9 | N |
| pdb_00003doh | N |
| pdb_00003guu | N |
| pdb_00003hea | N |
| pdb_00003hi4 | N |
| pdb_00003i6y | N |
| pdb_00003ia2 | N |
| pdb_00003s8y | N |
| pdb_00003t4u | N |
| pdb_00003t52 | N |
| pdb_00003vis | Y |
| pdb_00003wj1 | N |
| pdb_00003wyn | Y |
| pdb_00003zpx | N |
| pdb_00004cg3 | Y |
| pdb_00004eb0 | Y |
| pdb_00004ftw | N |
| pdb_00004opm | N |
| pdb_00004rnc | N |
| pdb_00004uhc | N |
| pdb_00004uhd | N |
| pdb_00004uhe | N |
| pdb_00004uhf | N |
| pdb_00004uyu | N |
| pdb_00005dwd | N |
| pdb_00005egn | N |
| pdb_00005frd | N |
| pdb_00005h6b | N |
| pdb_00005lui | Y |
| pdb_00005luk | Y |
| pdb_00005lul | Y |
| pdb_00005xjh | Y |
| pdb_00005zoa | Y |
| pdb_00006ane | Y |
| pdb_00006eqd | Y |
| pdb_00006eqe | Y |
| pdb_00006eqf | Y |
| pdb_00006eqg | Y |
| pdb_00006eqh | Y |
| pdb_00006h0t | N |
| pdb_00006h0v | N |
| pdb_00006i8w | N |
| pdb_00006ij3 | Y |
| pdb_00006ij5 | Y |
| pdb_00006ij6 | Y |
| pdb_00006ilw | Y |
| pdb_00006ilx | Y |
| pdb_00006kuo | Y |
| pdb_00006kuq | Y |
| pdb_00006kus | Y |
| pdb_00006nkg | N |
| pdb_00006qgc | Y |
| pdb_00007cef | Y |
| pdb_00007cqb | Y |
| pdb_00007ctr | Y |
| pdb_00007osb | Y |
| pdb_00007qjo | Y |
| pdb_00007qjq | Y |
| pdb_00007qjr | Y |
| pdb_00007qjs | Y |
| pdb_00007qvh | Y |
| pdb_00007vwn | Y |
| pdb_00007w1n | Y |
| pdb_00007w45 | Y |
| pdb_00007xjp | N |
| pdb_00007yko | Y |
| pdb_00007ykp | Y |
| pdb_00007ykq | Y |
| pdb_00008cmv | Y |
| pdb_00008cru | Y |
| pdb_00008d1d | Y |
| pdb_00008gu4 | Y |
| pdb_00008gu5 | Y |
| pdb_00008gzd | Y |
| pdb_00008h5k | Y |
| pdb_00008h5l | Y |
| pdb_00008h5o | Y |
| pdb_00008h83 | Y |
| pdb_00008j17 | Y |
| pdb_00008j45 | Y |
| pdb_00008j5n | Y |
| pdb_00008k7p | N |
| pdb_00008ota | Y |
| pdb_00008qrj | Y |
| pdb_00008spk | Y |
| pdb_00008vce | N |
| pdb_00008ve9 | Y |
| pdb_00008vek | Y |
| pdb_00008vel | Y |
| pdb_00008vem | Y |
| pdb_00008yl6 | N |
| pdb_00008yn1 | N |
| pdb_00008ytu | Y |
| pdb_00008ytv | Y |
| pdb_00009ewr | Y |
| pdb_00009jca | N |
| pdb_00009jcb | N |
| pdb_00009jux | N |
| pdb_00009kl9 | Y |
| pdb_00009lf7 | N |
| pdb_00009lj7 | Y |
| pdb_00009lmt | Y |
| pdb_00009lmu | Y |
| pdb_00009lmw | Y |
| pdb_00009m8h | Y |
| pdb_00009o9w | Y |
| pdb_00009pm1 | Y |
| pdb_00009pm2 | Y |
| pdb_00009qyp | Y |
| pdb_00009qyt | Y |
| pdb_00009rd2 | Y |
